# Gene Expression Profile in Peripheral Blood Nuclear Cells of Small Ruminant Lentivirus-Seropositive and Seronegative Dairy Goats in Their First Lactation

**DOI:** 10.3390/ani11040940

**Published:** 2021-03-26

**Authors:** Joanna Pławińska-Czarnak, Alicja Majewska, Joanna Zarzyńska, Janusz Bogdan, Jarosław Kaba, Krzysztof Anusz, Emilia Bagnicka

**Affiliations:** 1 Department of Food Hygiene and Public Health Protection, Institute of Veterinary Medicine, Warsaw University of Life Sciences, Nowoursynowska 159, 02-776 Warsaw, Poland; joanna_zarzynska@sggw.edu.pl (J.Z.); janusz_bogdan@sggw.edu.pl (J.B.); krzysztof_anusz@sggw.edu.pl (K.A.); 2 Department of Physiology Sciences, Institute of Veterinary Medicine, Warsaw University of Life Sciences, Nowoursynowska 159, 02-776 Warsaw, Poland; alicja_majewska@sggw.edu.pl; 3 Division of Epidemiology and Veterinary Management, Institute of Veterinary Medicine, Warsaw University of Life Sciences, Nowoursynowska 159c, 02-776 Warsaw, Poland; jaroslaw_kaba@sggw.edu.pl; 4 Institute of Genetics and Animal Biotechnology, Polish Academy of Sciences, Postepu 36A, Jastrzebiec, 05-552 Magdalenka, Poland; e.bagnicka@igbzpan.pl

**Keywords:** goat, peripheral blood nuclear cells, small ruminant lentivirus, microarray, gene expression, GPR37, GIMAP2, SSC5D, SETX

## Abstract

**Simple Summary:**

Caprine arthritis encephalitis, caused by small ruminant lentivirus (SRLV), is a disease that develops with various signs in adult goats, e.g., arthritis, mastitis, and progressive weight loss, while in goat kids, the disease presents with only neuropathy and extremely rarely. The disease results in reduced milk production and economic losses in herds of goats. Previously described changes in single gene expression do not fully explain all the processes occurring in the infected goats. Therefore, the present study describes the first use of a transcriptomic array designed specifically for goats in Poland. Its aim was to investigate the gene expression profiles of peripheral blood nuclear cells from SRLV-seropositive and SRLV-seronegative goats using a custom-made *Capra hircus* gene expression array. Just four genes out of ~50,000 were found to have differential expression; moreover, changes in their expression suggest an active inflammatory mechanism in SRLV-seropositive goats at the early stage of SRLV infection.

**Abstract:**

The immune response to a viral antigen causes inflammatory cell infiltration to the tissue, which creates a suitable environment for the replication of the virus in macrophages, and the recruitment of more monocytes to the site of infection, or latently infected monocytes. The aim of the study was to analyze the transcriptomic profile of peripheral blood nuclear cells isolated from SRLV-seropositive and SRLV-negative goats at the peak of their first lactation. SRLV-seropositive goats were probably infected via colostrum. Custom transcriptomic microarrays for goats were designed and developed, namely the *Capra hircus* gene expression array, which features ~50,000 unique transcripts per microarray. Only four genes were differentially expressed, with up-regulated expression of the GIMAP2, SSC5D and SETX genes, and down-regulated expression of the GPR37 gene in SRLV-seropositive vs. SRLV-seronegative goats. However, in an RT-qPCR analysis, the result for the SETX gene was not confirmed. The differences in the expressions of the studied genes indicate an active inflammatory process in the SRLV-seropositive goats at the early stage of infection.

## 1. Introduction

Small ruminant lentivirus (SRLV) belongs to the Retroviridae family. This is the single-stranded RNA virus. It causes caprine arthritis encephalitis in goats and maedi-visna in sheep [1]. Caprine arthritis encephalitis (CAE) is a chronic disease that is particularly prevalent in herds of dairy goats. CAE was first described in the United States in 1974 [2]. Since then, CAE has been reported all over the world, and its presence was confirmed in Poland in 1996 [3,4]. The SRLV can be easily transmitted from infected does to their suckling kids via colostrum and milk. Horizontal transmission can also occur through water and air exhaled from infected animals, although this requires long direct and indirect contact between goats [5,6]. Nowicka [7] reports that SRLV-seropositive bucks can encourage the spread of SRLV infection in goat herds.

According to Blacklaws [8], natural infection of SRLV takes place as a result of infiltration into dendritic cells of the mucous membranes of the respiratory tract or intestine by the virus, with cells then migrating to nearby lymph nodes. The virus infects monocytes and, using reverse transcriptase enzymes, forms a provirus, whereby DNA is integrated into the host genome. Infected macrophages can leave the lymph node and cause systemic infection. Persistent infection occurs through the infection of bone marrow cells, myeloid stem cells, or myeloid precursors [9]. Infected monocytes migrate to other tissues, e.g., those of the udder and lung, where they differentiate into tissue macrophages. As this process is very slow, the disease likewise develops slowly, with clinical symptoms manifesting as late as 12 months after infection [4]. Unlike most other members of the genus, such as the human, simian, or feline immunodeficiency viruses (HIV, SIV, and FIV, respectively), SRLV is unable to infect lymphocytes [10]. This difference is probably why immunosuppression and opportunistic infections are not hallmarks of lentiviral infections in small ruminants. However, the fact that the expression of some cytokines and acute phase proteins in blood leukocytes differ between SRLV-seropositive (SRLV-SP) and SRLV-negative (SRLV-SN) goats suggests that the virus does impact immunity [11,12,13].

SRLVs produce lifelong infections, which can remain dormant for a long time; however, eventually the virus elicits clinical and pathological symptoms. There are several clinical presentations, such as polyarthritis and induration of the udder with hypogalactia. Chronic interstitial pneumonia and wasting syndrome can also occur, with chronic progressive polyarthritis being the most common [14]. Neurological symptoms are observed only in goat kids, and they are quite rare [15]. Immune activation, including the response to the viral antigen, causes inflammatory cell infiltration into the tissue. This response creates a suitable environment for the replication of the virus in macrophages, the recruitment of more monocytes to the infection site, or greater latent infection in monocytes [8]. Several studies have explored the influence of the infection on the productivity of affected herds [12,16]. In some studies, SRLV infection seems to reduce milk yield, but these studies considered only uniparous or multiparous does. Nowicka et al. [7] report that fat and protein contents were also reduced, but only if allowance of the supplementary feed was low.

The occurrence of individual clinical symptoms depends on the genetic factors of the virus and the host organism. Our comparative studies between SRLV-SN and SRLV-SP goats in terms of milk yield and changes in basic milk production parameters revealed a decrease in the amount of milk secreted and an increase in the number of somatic cells in the milk of SRLV-SP goats [17]. This finding was particularly evident in goats in their first and second lactations and is probably related to the multiplication and spread of the virus in the mammary gland, as well as the destruction of galactopoietic cells in the developing mammary gland. We hypothesized that due to the nature of the lentiviral infection, the virus could influence the regulation of gene expression.

As mentioned above, some studies on the influence of SRLV infection on immune-related genes have been conducted. Decreased expressions of IL-1α, IL-1β, IL-6, and IFN-γ were found, in addition to increased TNFα expression in the blood leukocytes of the SRLV-seropositive goats [11,18]. Moreover, higher expression levels of the SAA and Hp genes have been found in blood leukocytes of SRLV-seropositive animals [12]. However, information about the influence of SRLV infection on gene expression is still limited, and further studies are needed to fully understand the processes occurring in infected goats. Previous studies have determined differences in gene expression between two major Polish goat breeds, PWI and PFI. When the transcriptomic profiles of SRLV-SN PFI and PWI goats were compared, significant changes were found in the expression of the gene encoding the *Capra hircus* agouti signaling protein (ASIP), which is responsible for the color of the goat (*p* ≤ 0.05). In that study, a hierarchical grouping of transcriptomic profiles of three biological materials (milk somatic cells, milk fat globules, and PBNCs) from these two goat breeds showed three different subgroups of clusters corresponding to the examined material, which confirmed that the main factor causing the clustering was the origin of the sample, not the breed of the goat [19].

Taking this into account, the aim of this study was to investigate the gene expression profiles of peripheral blood nuclear cells (PBNCs) from SRLV-SP and SRLV-SN goats of two Polish goat breeds using a custom-made *Capra hircus* gene expression array.

## 2. Materials and Methods

### 2.1. Animals

Two Polish goat breeds were chosen for this study: Polish White Improved (PWI) and Polish Fawn Improved (PFI). Four SRLV-SN goats (*n* = 4) and four SRLV-SP goats (*n* = 4) in the peak of the 1st lactation (70–80 day of lactation) were used in the experimental group, while seven SRLV-SN goats in their fourth lactation were used as reference group animals (PWI *n* = 4 and PFI *n* = 3). The animals were born and maintained in the Experimental Farm of the Institute of Genetics and Animal Biotechnology in Jastrzębiec, Poland (IGAB). The animals were kept in two groups (i.e., the SRLV-SN goats were kept separately from SRLV-SP goats), in separate pens, where water and hay were given ad libitum. Maize silage with concentrates was supplied according to the average milk yield of the goats in the group. In the summer period, the diet was supplemented with green forage, by grazing on pasture. The goats were fed according to the system developed by the Institut National de la Recherche Agronomique (INRA) of France and adopted by the Research Institute of Animal Production Poland [20]. After the study, the goats were maintained in the experimental farms’ (IGAB) productive system.

### 2.2. Serodiagnosis of SRLV

SRLV infection was confirmed or ruled out based on the results of ELISA analysis of serum samples from animals selected for the research groups. All serological examinations were performed using two ELISA tests (ID Screen MVV/CAEV Indirect-Screening test, ID.vet, Grabels, France and IDEXX CAEV/MVV Total Ab Screening Test, IDEXX Laboratories, Inc., Westbrook, ME, USA) [21]. The tests have been conducted regularly every six months in this herd for 20 years, with the first test for the animal occurring at the age of six months. During the study, tests were also performed twice a year to identify new potential infections and to eliminate infected animals from the control group. The presence of the virus in the herd was also confirmed by its isolation [22]. All goats were asymptomatic: none demonstrated any sign of CAE, regardless of serological status.

The goats were under the constant care of a veterinarian, and animals with clinical signs of any other diseases were excluded from the study. At the same time, each goat involved in the study was clinically examined by a specialist who was a Diplomate of the European College of Small Ruminant Health Management (coauthor JK). As the first antibodies may appear as late as one year after the onset of infection [21], the samples for microarray analysis were collected from all goats at the peak of their first lactation, e.g., at the age of 1.5 years. The group of SRLV-SN animals was isolated from their mothers after being born, prior to colostrum collection, and only those that showed no seroconversion until the end of the studied lactation were included. The SRLV-SP group received colostrum from their SRLV-SN mothers and only included goats that were determined to be SRLV-SP by ELISA at the age of six months, as well as in the subsequent tests conducted every six months. The does included in the SRLV-SN group were separated from their mothers, regardless of the mother’s serological status, and fed cow’s colostrum for the first five days followed by milk replacer (Sprayfo Primo Goat Kid, Trouw Nutrition, Grodzisk Mazowiecki, Poland) [23]. The SRLV-SN goats were milked first to eliminate the risk of SRLV transfer via milking equipment.

### 2.3. Blood Sampling and RNA Isolation

Blood was collected from the animals between day 70 and day 80 of lactation, and RNA was extracted from nucleated blood cells according to the procedures described by Pławińska-Czarnak [19]. Total RNA was isolated from blood samples using a Total RNA Mini Kit (A&A Biotechnology, Gdynia, Poland), according to the manufacturer’s protocol.

#### RNA Quality Assessment

The quantity and purity of RNA were determined by NanoDrop spectrophotometer (NanoDrop Technologies, Wilmington, DE, USA). The purity of total RNA was confirmed by absorbance ratio A260/A280 values ranging between 2.01 and 2.07. RNA quality and integrity were analyzed using an Agilent 2100 Bioanalyzer (USA) and an RNA 6000 Nano Kit (Agilent Technologies, Waldbronn, Germany). Total quality RNA was rated using an RNA Integrity Number (RIN) generated by Agilent Software Expert. All information and sample data were collected in a custom database, described by Pławińska-Czarnak et al. [24]. Only RNA samples with an RIN ≥ 8.5 were included in the analysis.

### 2.4. Microarray Analysis

The experiment was performed using a common reference design: the common reference comprised a pool of equal amounts of RNA from seven SRLV-SN goats of both Polish breeds (PWI, *n* = 4 and PFI, *n* = 3) in their fourth lactation. These RNA samples were not used as the experimental samples in the microarray analysis. As was mentioned above, experimental samples were collected from SRLV-SN and SRLV-SP goats at the peak of their first lactation (*n* = 4 in each group). RNA was isolated from PBNCs.

Two-color microarrays were used. On each microarray, 300 ng of a cRNA Cy3-labeled common reference sample and 300 ng of a cRNA Cy5-labeled SRLV-SN goat or an SRLV-SP goat (each from a single goat) were hybridized.

The gene expression profile analysis was performed using a SurePrint G3 Goat Gene Expression Microarray, 8 × 60 K, with approximately 50,000 transcripts on each microarray (Agilent Technologies, Santa Clara, CA, USA) and an Agilent Technologies Reagent Set, according to the manufacturer’s instructions. A RNA Spike-In Kit was used as an internal control, and a Low Input Quick Amp Labeling Kit was applied to amplify and label (as Cy3 or Cy5) the target RNA to generate complementary RNA (cRNA) for oligo-microarrays. A Gene Expression Hybridization Kit (Agilent Technologies) was used for fragmentation and hybridization, and a Gene Expression Wash Buffer Kit (Agilent Technologies) was used to wash the slides after hybridization. Hybridization intensities were acquired and analyzed using an Agilent DNA Microarray Scanner G2505C.

### 2.5. Signal Detection and Statistical Analysis

After microarray scanning, the data were extracted and the background subtracted according to the standard procedures contained in the Agilent Feature Extraction (FE) software, version 10.7.3.1. FE performs Lowess normalization.

The statistical analysis was performed using Gene Spring software ver. 14.9.1 (Agilent Technologies). The statistical significance of the differences was evaluated using moderated *t*-test with a corrected *p*-value cut-off of 0.05, and a multiple testing correction was performed using a Benjamini and Hochberg False Discovery Rate (FDR) < 5%. Microarray data were deposited in the Gene Expression Omnibus data repository under the number GSE121259.

### 2.6. Validation of Microarray Data

Reverse transcription-quantitative PCR (RT-qPCR) was used to confirm the microarray results. The expressions of the following four genes were validated: *Capra hircus* GTPase, IMAP family member 2 (*GIMAP2*)*, Capra hircus* G protein-coupled receptor 37 (*GPR37*)*, Capra hircus* scavenger receptor cysteine-rich family member with five domains (*SSC5D*), and *Capra hircus* senataxin (*SETX*).

The sequences of the chosen genes were obtained from Ensembl or the NCBI database. Primers were designed using Primer-Blast (free software available online provided by NCBI, U.S. National Library of Medicine) and verified using Oligo Calc: Oligonucleotide Properties Calculator [25,26] to exclude sequences showing self-complementarity. The secondary structures of the amplicons were examined using the m-fold web server [27]. The two reference genes: 18S ribosomal RNA (18S rRNA—protein synthesis) and ribosomal protein large, P0 (*RPLP0*) were chosen based on previously-published results [28]. Additionally, the stability of the reference genes was validated using the GeNorm and NormFinder programs. All primer sequences are listed in Table 1. The RT-qPCR reaction was performed in triplicate using SYBR Select Master Mix (Applied Biosystems, Beverly, MA, USA) on a Stratagene Mx3005P Quantitative PCR instrument (Agilent Technologies), according to the manufacturer’s protocol. The conditions of the reactions were established according to the manufacturer’s protocol and comprised the following steps: polymerase activation at 95 °C for two minutes, then 40 cycles of amplification (denaturation at 95 °C for 15 s, annealing at 58 °C for 15 s, and extension at 72 °C for one minute).

All measurements were collected in triplicate for each biological sample and the mean value was used for further calculations. Results were calculated using the 2^−ΔΔCT^ method [29].

To identify signaling pathways and gene function, the microarray data were analyzed using Pathway Studio 12.4.0.3 [30].

## 3. Results

The results of the microarray analysis revealed significant changes in the expression of only four of approx. 50 thousands genes: namely, *GIMAP2, SSC5D,* and *SETX* demonstrated higher expression in SRLV-SP goats than in SRLV-SN goats, whereas *GPR37* demonstrated lower expression in the SRLV-SP goat group (Table 2). RT-qPCR validation of these showed significant changes in expression were observed for *GIMAP2, GPR37,* and *SSC5D*, with the same direction of changes; however, no significant difference was observed for *SETX*. These results are presented in Table 2.

The activity of the protein products of the *GPR37*, *GIMAP2*, and *SSC5D* genes has been described in relation to the immune response in humans and other animals.

**Figure 1:**
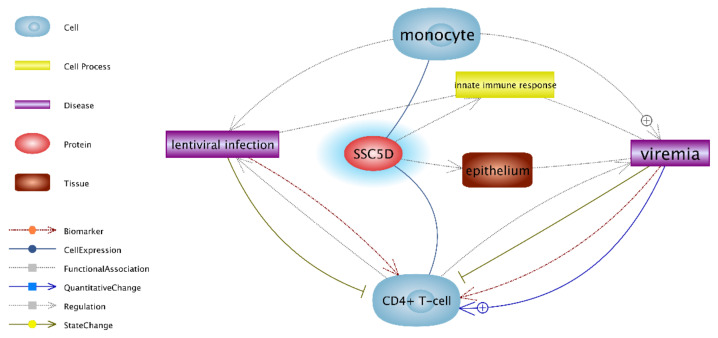
Direct links between the SSC5D protein and monocytes and CD4^+^ T cells during lentivirus infection. Upregulated expression of the *SSC5D* gene in PBNCs of SRLV-SP vs. SRLV-SN goats, as analyzed by the Pathway Studio program [30]. The blue line highlights the upregulation of the gene in SRLV-SP goats vs. SRLV-SN goats. SSC5D—scavenger receptor cysteine-rich family member with five domains (alias S5D-SRCRB); PBNCs—peripheral blood nuclear cells; RT-qPCR—quantitative reverse transcription PCR; SRLV-SP—small ruminant lentivirus-seropositive; SRLV-SN—small ruminant lentivirus-seronegative.

As presented in Figure 1, *SSC5D* demonstrates high expression in monocytes and CD4^+^ T-cells during the early stages of SRLV-infection regulating the innate immune response.

**Figure 2:**
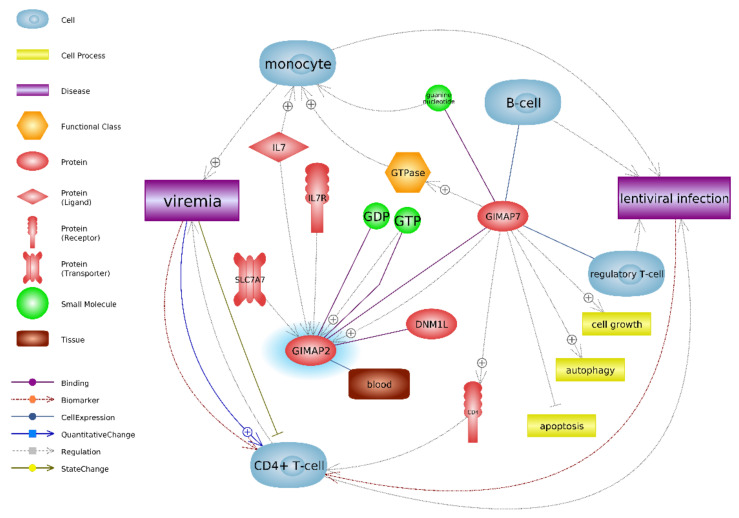
Interactions between proteins, cells, and lentiviral infection for the *GIMAP2* gene in PBNCs, as analyzed by the Pathway Studio program [30]. Blue highlights upregulation in SRLV-SP goats vs. SRLV-SN goats; GIMAP2—GTPase, IMAP family member 2; GIMAP7—GTPase, IMAP family member 7 (GIMAP7 -> GIMAP2 can stimulate both its own GTPase activity and that of GIMAP2 by dimerization and the provision of a catalytic arginine finger); DNM1L—dynamin-1-like protein (gene encodes a member of the dynamin superfamily of GTPases); SLC7A7—solute carrier family 7 member 7 (regulates 11 gene expression signature genes, including GIMAP2, IL10RA, and IL18as); IL7R—interleukin 7 receptors (->Regulation IL7R->GIMAP2; the signal given by IL-7/IL-7R affects the regulation of apoptosis, cell cycle progression, and differentiation, cell type CD4^+^ T cell); GDP—guanosine diphosphate; GTP—guanosine triphosphate; IL7—interleukin 7; CD4^+^ T-cell—T-cell surface glycoprotein CD4 precursor (this gene encodes a membrane glycoprotein of T lymphocytes that interacts with major histocompatibility complex class II antigens and is also a receptor for the human immunodeficiency virus).

The protein product of the *GIMAP2* gene also interacts with monocytes and CD4^+^ T-cells; however, not directly. Its expression is regulated by innate immune agents such as Il-7 and Il-7R, as well as GIMAP7: another member of the IMAP family (Figure 2). Both proteins SSC5D and GIMAP2 are directly (SSCD5) or indirectly (GIMAP2) involved in the cellular response of the monocyte/macrophage lineage in lentiviral infection, and their activity could be connected with viremia.

**Figure 3:**
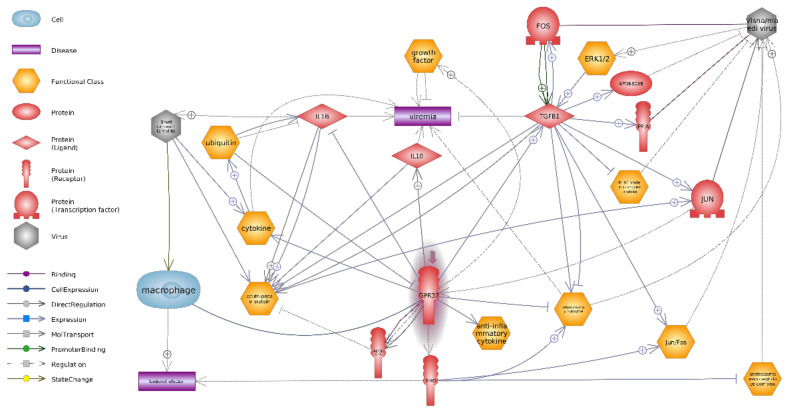
Involvement of GPR37 in processes related to the cellular response to infection with small ruminant lentiviruses/visna-medi virus in goats. Pink highlights downregulation; GPR37—G protein-coupled receptor 37; FOS-Fos proto-oncogene, (AP-1 transcription factor subunit) is a protein coding gene. These genes encode leucine zipper proteins that can dimerize with proteins of the JUN family, thereby forming the AP-1 transcription factor complex. The interaction between Fos and Jun targets the Tat visna virus and promotes its replication in monocytes and macrophages after their differentiation. Fos-Jun activation can influence the activation of many cellular genes; this could intensely affect cell and viral growth and thus play a role in the pathogenesis of the visna virus; TGFB1—*TGFβ*1 gene encodes a secreted ligand of the TGF-beta (transforming growth factor-beta) superfamily of proteins. This protein regulates cell proliferation, differentiation, and growth, and can modulate the expression and activation of other growth factors, including interferon gamma and tumor necrosis factor alpha [31]. IL1B-interleukin 1β; IL-10 interleukin 10; APOBEC3B—apolipoprotein B mRNA editing enzyme catalytic polypeptide-like 3. The antiviral APOBEC3 protein is packaged into the forming virions and limits replication by causing mutations in viral cDNA. PPIA (aliases CYPA, Cyclophilin A)—the *PPIA* gene encodes a member of the peptidyl-prolyl cis-trans isomerase (PPIase) family. In goat SRLV, *Vif* can exploit the host cellular PPIA protein as a co-factor for caprine APOBEC3 degradation. [32]; SMO (Smoothened). The *SMO* gene encodes a member of the Frizzled family of G protein-coupled receptors, which is connected with the hedgehog (Hh) signaling pathway. In humans, SMO inhibits infection by HIV-1 and limits the spread of HIV-1 infection by suppressing the formation of viral DNA [33].

The downregulation of the *GPR37* gene (Figure 3), visible in the pathway analysis, seems to be due to extracellular cues and the activation of intracellular signal transduction pathways.

**Figure 4:**
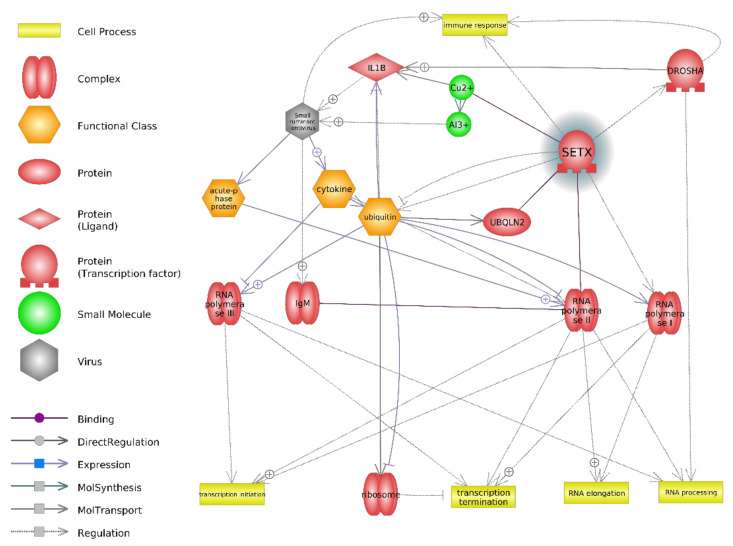
Interactions between proteins, cell process, and lentiviral infection for the protein product of the *SETX* gene in PBNCs. DROSHA—gene encoding a ribonuclease (RNase) III double-stranded RNA-specific ribonuclease. IL1B—interleukin 1β; IgM—Immunoglobulin M.

Although the microarray result was not confirmed by RT-qPCR in the case of the *SEXT* gene, links were observed between small ruminant lentivirus infection and SEXT regarding certain cellular processes, such as transcription process, immune response, and transcription termination (Figure 4). The pathways presented are similar to the changes occurring in HIV-1 infection (Figure S1).

**Figure 5:**
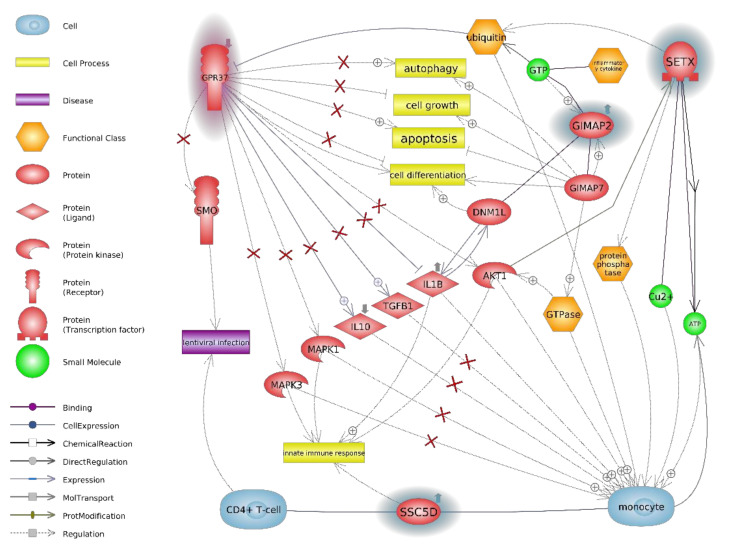
The interactions between proteins, cells, and lentiviral infection for GPR37 in connection with SSC5D, GIMAP2, and SETX on the *Capra hircus* microarray. Blue highlights upregulation; pink highlights downregulation; GPR37—G protein-coupled receptor 37; SMO—Smoothened, seven transmembrane spanning receptor; MAPK3—mitogen-activated kinase 3; IL1B—interleukin 1β; IL10—interleukin 10; AKT1—AKT serine/threonine kinase 1; TGFB1—transforming growth factor β-1; MAPK1—mitogen-activated protein kinase 1; DNML—dynamin-1-like protein; SETX—senataxin; SSC5D—scavenger receptor cysteine-rich family member with five domains; GDP—guanosine diphosphate; GTP—guanosine triphosphate; GIMAP2—GTPase, IMAP family member 2; GIMAP7—GTPase, IMAP family member 7 (GIMAP7 -> GIMAP2 can stimulate both its own GTPase activity and that of GIMAP2 by dimerization and the provision of a catalytic arginine finger). Low *GPR37* expression de facto increases the expression of pro-inflammatory cytokines (e.g., IL-1β) which favors the maturation of monocytes to an M1 macrophage phenotype; however, it also suppresses the expression of anti-inflammatory cytokines (e.g., IL-10) in macrophages and delimits polarization to an M2 macrophage phenotype [34].

The links between all genes were studied in detail (Figure 5). The proteins coded by the *GPR37*, *SSC5D, GIMAP2*, and *SETX* genes are involved in various cellular processes, as well as in several diseases, including lentiviral infection. As the goats used in the study were 1.5 years old, and their somatic development was not finished, it is not surprising that the identified cellular processes included cell growth and differentiation. However, changes in autophagy and apoptosis processes could be associated with virus elimination.

## 4. Discussion

The immune response to lentivirus infection is a complex and dynamic process, and so far, little has been uncovered about how the immune system responds to these infections in goats. Nevertheless, the topic remains the subject of much research [11,12,18,21,35].

Our findings indicate that, of approximately 50,000 studied transcripts, only three genes (*GPR37*, *GIMAP2*, and *SSC5D*) demonstrated different expression in goat PBNCs at the peak of the first lactation as a result of SRLV infection. This is a surprisingly low number, as microarray studies typically reveal changes in the expression of several hundred or even thousands of genes, and some of these are then selected to verify the microarray using RT-qPCR [36,37,38]. The *SSC5D* gene codes for soluble scavenger receptor cysteine-rich domain-containing protein (SSC5D), which binds to extracellular matrix proteins. Gonçalves et al. [39] suggest that *SSC5D* has restricted expression, being exclusively expressed by monocytes/macrophages and T lymphocytes. Moreover, it is possible that SSC5D is active at the interface of innate and adaptive immunity. An expression analysis of lymphoid cells revealed that SSC5D is primarily expressed in monocytes and also in T lymphocytes, but is absent in B lymphocytes. Monocytes/macrophages, as the main targets of SRLV, play a key role in the infection process, spreading the virus and providing antigens, costimulatory signals, and cytokines, these being inducers or regulators of the immune response. SRLV infects monocyte-line cells in the bone marrow of a susceptible host. Circulating monocytes do not show a high degree of replication of the virus: it is only after their maturation into macrophages that viral replication begins. The main target tissues include those of the mammary gland, synovia, lung, and central nervous system [40]. Monocyte/macrophage line cells are heterogeneous, reflecting the plasticity and versatility of responses to environmental stimuli required for effective immune defense. The classic macrophage (M1 phenotype) and alternative activation (M2 phenotype) depend on the presence of molecules secreted by CD4^+^ T cells [41]. Examples of direct links between the SSC5D protein and monocytes and CD4^+^ T cells during lentivirus infection are shown in Figure 1.

The *GIMAP2* (GTPase, IMAP Family Member 2) gene encodes GTPases of immunity-associated proteins (GIMAPs). *GIMAP* genes are grouped in chromosomal clusters and are abundantly expressed in cells of the immune system. GIMAPs are a distinct family of GTPases that controls apoptosis in lymphocytes and plays a central role in lymphocyte maturation and lymphocyte-associated diseases [42]. However, until now, there have been no scientific reports on the involvement of these genes, or the relationship between them, on the inflammation caused by the lentivirus in goats. Although Kaba et al. [15] report differences in the subpopulations of T lymphocytes, viz. CD4^+^, CD8^+^, TCR1-N6, WC1-N2, and WC1-N3, in goats chronically infected with SRLV [15] it should be stressed that unlike viruses such as HIV-1, SRLV does not infect lymphocytes [8]. Nevertheless, *GIMAP* genes have been described in the context of many diseases in humans and, importantly, are associated with the early cellular response induced by RNA viruses, including lentiviruses [43].

Studies on the gene expression profile of HCV (Hepatitis C Virus)-infected liver fibrosis in humans found *GIMAP2* to be upregulated in the early stage of the disease as compared to the later stages [44]. A study of the transcriptomic and epitranscriptomic landscape of HIV-infected cells (SupT1 cells: a CD4^+^ T-cell-line) by Cristinelli et al. [42] revealed the presence of four out of the seven GTPase immuno-associated proteins (*GIMAP*) included GIMAP2.

As was mentioned above, differences have been found between the subpopulations of T lymphocytes in goats chronically infected with SRLV. The percentage of whole subpopulations of T lymphocytes was higher in SRLV-SP goats than in SRLV-SN goats [15]. However, Ponti et al. [45] report elevated levels of CD8^+^ lymphocytes with no concurrent change in CD4^+^ cells, suggesting that changes may be characteristic of an early stage of the infection in goats. Further research is needed regarding the possible linkage of the increased GIMAP2 expression in blood leukocytes in goats in the early stages of SRLV infection.

Unlike GIMAPs, SRLV has been found to influence the expression of the *SSC5D* gene in T lymphocytes, and trigger *GIMAP2* gene transcription in the spleen, lymph nodes, thymus, and leukocytes [44]. In turn, GIMAP2 plays a crucial role in lymphocyte maturation at the early stage of infection.

The most surprising result was the change in expression of the *GPR37* gene. Its expression was significantly reduced in SRLV-SP goats relative to SRLV-SN goats (Table 1). *GPR37* codes for the G protein-coupled receptor 37 protein, which contains seven transmembrane domains and is found in cell and endoplasmic reticulum membranes. G protein-coupled receptors are involved in translating outside signals into G protein-mediated intracellular effects [46]. To date, the *GPR37* gene has been studied and widely described in Parkinson’s disease [47,48,49,50], in the central nervous system [51,52], and in multiple cancers [53,54,55] and also in various mouse tissues [56]. Bang et al. [57] found GPR37 to regulate macrophage phenotypes, such as the proinflammatory M1 phenotype and anti-inflammatory M2 phenotype; they also indicate that in macrophages, GPR37 regulates phagocytosis in inflammatory processes of mouse skin. GPR37-expressing macrophages may contribute to the reduction of inflammatory pain [58].

Qu et al. [34] report that a decrease in *GPR37* expression results in increased expression of pro-inflammatory cytokines (e.g., IL-1β) and mutual suppression of the expression of anti-inflammatory cytokines in macrophages; for example, it suppresses IL-10, which participates in the differentiation of cells into M2 macrophages [34] (Figure 3). This suggests that low expression of *GPR37* promotes the M1 phenotype. Jarczak et al. [11] found both decreased expression of *IL-1β* at both mRNA and protein levels in blood leukocytes/serum of SRLV-seropositive, asymptomatic goats; however, no differences in *Il-10* mRNA level or concentration were observed between the 2nd and upper 3rd lactation [11]. Nevertheless, GPR37 deficiencies cause dysregulation of pro-inflammatory and anti-inflammatory cytokines and a decrease in macrophage activity [34]. Therefore, the decreased expression of *GPR37* found in our study may be associated with the inhibition of monocyte/macrophage apoptosis and the promotion of the spread of the provirus to the marrow and then to the target tissues. Our present findings and those of Jarczak et al. [11] indicate that downregulation of *GPR37* gene expression occurs only at the early stage of infection.

Liu et al. [53] report that *GPR37* knockdown induced S phase arrest, with a concomitant reduction in the number of cells in the G1 phase compared to control siRNA, implying that *GPR37* can curtail the G1-S transition of the cell cycle, and thus cell growth. Transient knockdown of *GPR37* by siRNA in HuH7 cells significantly reduced apoptosis in hepatoma cells, with activation of the *3-Akt* phosphatidylinositol signaling pathway (Figure 3). Relatively few studies have examined the actions of *GPR37* during its low activity. It may be possible that a similar mechanism of limiting apoptosis occurs in macrophages infected with SRLV, thus promoting their spread in the organism. Our findings indicate decreased expression of *GPR37*, which may mean that low levels of its protein products influence macrophage phenotypes by failing to suppress the expression of pro-inflammatory cytokines (IL-1β) and preventing the increased expression of anti-inflammatory cytokines (IL-10 and TGF-β) [57].

It seems that GPR37 may play an important role in the macrophage polarization process in the first stage of SRLV infection. Jarczak et al. [18] found that goats infected with SRLV for many years demonstrated lower expression of *IFN-γ, IL1α, IL-1β,* and *IL-6* mRNA and higher expression of *TNFα* in blood leukocytes. At the protein level, they report lower concentrations of IL1α and IL-1β in the serum of infected goats. However, certain methodological differences exist between these two studies: for example, one uses goats infected for many years (and were at least three-years-old) while the present study uses goats in the early stage of infection, and in their first lactation; however, in all cases, none of the goats had any sign of CAE. Figure 3 shows the importance of the direction of regulation of the *GPR37* gene affecting the production of pro-inflammatory and anti-inflammatory cytokines.

Figure 5 shows the synergy of mutual interactions of the altered genes identified in the present study. All are involved in the body’s cellular response, which in the first stage, is associated with the spread of SRLV in the organism. The hierarchical arrangement of the *SSC5D*, *GIMAP2–GIMAP7* heterodimer, *GPR37,* and *SETX* genes, and their participation in the regulation of cellular processes (Figure S2).

*SETX* (senataxin) is also a protein-coding gene. Its protein product is known to play a role in transcription, neurogenesis, and antiviral response [59]. Suraweera et al. [60] report that SETX participates in transcriptional regulation through its ability to modulate RNA Polymerase II binding to chromatin and through its interaction with proteins involved in transcription [60]. The protein encoded by this gene contains a DNA/RNA helicase that may be involved in both DNA and RNA processing, and in diverse aspects of RNA metabolism and genomic integrity; for example, SETX plays a role in DNA repair and in the coordination of transcriptional events [61]. It is also involved in the response to DNA double-strand break damage generated by oxidative stress [62]. Vantaggiato et al. [63] described the function of SETX in neurite outgrowth in hippocampal cells through fibroblast growth factor 8-activated signaling pathways and in the inhibition of retinoic acid-induced apoptosis. SETX also plays a role in the development and maturation of germ cells, which is an essential process for male meiosis, acting at the interface of transcription and meiotic recombination [64].

Similar levels of *SETX* expression were observed in the SRLV-SN and SRLV-SP goats, suggesting that the 1.5-year-old goats are still in their development phase. *SETX* is known to be involved in a range of activities associated with RNA metabolism, genome integrity, DNA repair, and transcription coordination. As the protein product (SETX) attenuates viral transcription by forcing premature transcription termination, *SETX* can be regarded as an antiviral gene. In addition, TAF4, a transcriptional cofactor of *SETX*, has also been found to reduce transcription activation by premature transcription termination [59,65]; similarly, *SETX, XRN2*, and *RRP6/EXOSC10* are believed to act as cooperative genes to force premature termination of RNA transcription in HIV-1 infection [66,67] (Figure S1). However, the microarray result was not confirmed by RT-qPCR, and further study is needed to determine the processes connected to *SETX* activity. Changes in the immune response and the demonstrated disease processes observed between the two groups may have been the result of SRLV already beginning to exert its effect.

However, the present study included young animals that had been naturally infected approximately 1.5 years previously, when the animals had yet to show any clinical signs of the disease. It seems that the slow process of SRLV infection in these goats manifests by changes to the expressions of only a few genes. This phenomenon also confirms earlier findings that in the immune system of asymptomatic goats, the virus triggers changes in the expression of acute-phase proteins and cytokines at only a very low rate [11,12].

This study is the first to demonstrate that the *SSC5D*, *GIMAP2*, and *GPR37* genes present on the approx. 50,000 *Capra hircus* microarray are differentially expressed at the early stage of SRLV infection. Their targeted analyses indicate links between immune system elements and lentivirus infection. As these genes are likely connected to the innate response to viral infection, further study on the role of their protein products at the early stage of CAE in goats is needed.

In the future, the expression of these genes could be used as early markers of SRLV infection in young goats, up to 1.5 years of age. Furthermore, a comparative analysis of selected genes of SRLV infected goats both asymptomatic and with CAE sign could be recommended, as Bilbao-Arribas et al. [68] proposed in their miRNA studies in sheep.

## 5. Conclusions

In our study, only four genes from approximately 50,000 were found to be differentially expressed; however, the differences in their expressions indicate the presence of an active inflammatory process in the SRLV-SP goats at the early stage of SRLV infection. Moreover, targeted analyses of the *SSC5D*, *GIMAP2*, and *GPR37* genes indicate links between immune system elements and lentivirus infection. Despite the fact that SRLV does not infect lymphocytes, by modifying the expression of studied genes in leukocytes and in organs or glands, the virus can indirectly affect the activity of the protein products of these genes in the inflammation process caused by the lentivirus in goats. Protein products of the studied genes, especially SETX and GPR37, also appear to directly affect the processes related to somatic growth and development of 1.5-year-old goats.

## Figures and Tables

**Table 1:** Names, symbols, and NCBI accession numbers of genes and primers used in RT-qPCR verification of the microarray results.

Gene Name (Gene Symbol)	Forward	Reverse	NCBI Accession Number	Type of Gene/Source
	Primer (5′–3′)			
*Capra hircus* GTPase, IMAP family member 2 (*GIMAP2*)	GCTCATGGACTTGACTGAGA	ACAGCTTGGATTAGGGCTTT	XM_018047470.1	target
*Capra hircus* G protein-coupled receptor 37 (*GPR37*)	TGCAAGGAAAATCCGCAAAG	CAGAAGGAACTGGCTGATGA	XM_005679413.3	target
*Capra hircus* scavenger receptor cysteine-rich family member with 5 domains (*SSC5D*)	GACCTCACCTCAGCCTCTAT	GGTAGGTGGAGAGGTTACTTG	XM_018063058.1 XM_018063057.1	target
*Capra hircus* senataxin (*SETX*)	GGTAGACGGCTTTTCTTTCC	TCTTCTGCTTCCCAAGTTTCC	XM_018056034.1	target
Ribosomal protein large, P0 (*RPLP0*)	CAACCCTGAAGTGCTTGACAT	AGGCAGATGGATCAGCCA	NM_001012682.1	Reference gene [28]
18S ribosomal RNA *(18S rRNA)*	CAAATTACCCACTCCCGACCC	AATGGATCCTCGCGGAAGG	DQ_066896.1	Reference gene [28]

**Table 2:** Differentially expressed genes in the PBNCs of dairy goats at the peak of the first lactation (*n* = 8): a comparison between SRLV-SP and SRLV-SN goats.

Gene Name (Gene Symbol)	Biological Function	*Capra hircus* Microarray (Direction of Expression Changes)			RT-qPCR		
		Fold Change	Regulation	*p*-Value	Fold Change	Regulation	*p*-Value
*Capra hircus* GTPase, IMAP family member 2 (*GIMAP2*)	Protein binding GTP binding, identical protein binding	2.938	up	0.015	2.640	up	0.003
*Capra hircus* scavenger receptor cysteine-rich family member with 5 domains (*SSC5D*)	Fibronectin binding, scavenger receptor activity, protein binding, extracellular matrix binding	2.206	up	0.033	4.309	up	0.012
*Capra hircus* senataxin (*SETX*)	Nucleotide binding, transcription, termination site sequence-specific DNA binding, DNA binding, DNA helicase activity, helicase activity	3.193	up	0.015	1.06	up	0.780
*Capra hircus* G protein-coupled receptor 37 (*GPR37*)	G-protein coupled receptor activity, protein binding, G-protein coupled peptide receptor activity, Hsp70 protein binding	−13.305	down	0.015	−3.892	down	0.002

## Data Availability

Microarray data were deposited in the Gene Expression Omnibus data repository under the number GSE121259. https://www.ncbi.nlm.nih.gov/geo/query/acc.cgi?acc=GSE121259; (accessed on 24 March 2021). Agilent-083405 Genotypic_EA993_Goat_8x60k_array GPL25675. http://www.ncbi.nlm.nih.gov/geo/query/acc.cgi?acc=GPL25675; accessed on 24 March 2021.

## Supplementary Materials

The following are available online at https://www.mdpi.com/article/10.3390/ani11040940/s1. Figure S1: SETX as the main gene involved in the processes of termination of HIV-1 transcription, Figure S2: Hierarchical arrangement of the SSC5D, GIMAP2–GIMAP7 dimer, GPR37, and SETX genes and their involvement in the regulation of cellular processes.
