# Supplementary material for: Gene Expression Profile in Peripheral Blood Nuclear Cells of Small Ruminant Lentivirus-Seropositive and Seronegative Dairy Goats in Their First Lactation
Source: Animals (Basel). 2021 Mar 26;11(4):940. doi: 10.3390/ani11040940 (PMC8066113; doi:10.3390/ani11040940)
Supplement: Supplementary file 1 [file animals-11-00940-s001.pdf]

animals

MDPI

Supplementary Materials: Gene Expression Profile in Peripheral Blood Nuclear Cells of Small Ruminant Lentivirus-Seropositive and Seronegative Dairy Goats in Their First Lactation

Joanna Pławińska-Czarnak, Alicja Majewska, Joanna Zarzyńska, Janusz Bogdan, Jarosław Kaba, Krzysztof Anusz and Emilia Bagnicka

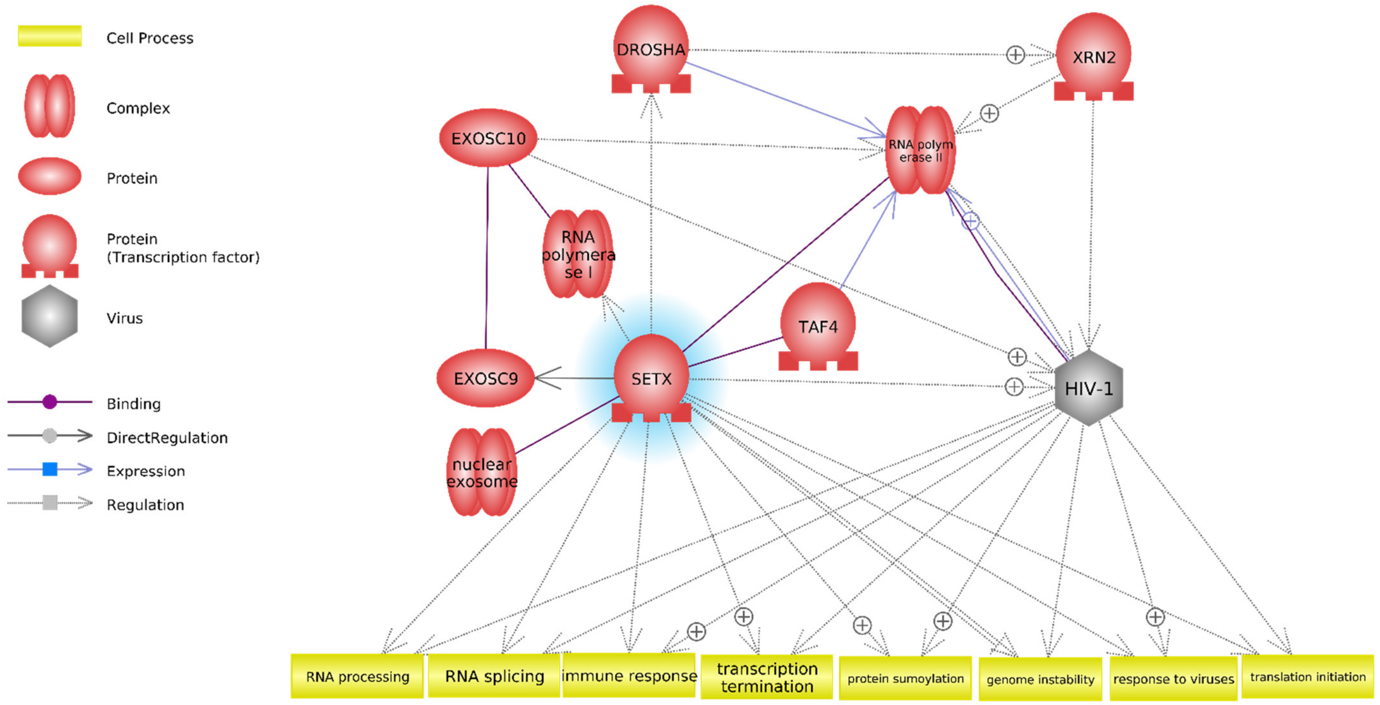

Figure 1. *SETX* as the main gene involved in the processes of termination of HIV-1 transcription. Schematic diagram showing connections between regulatory proteins, HIV-1 virus, and their joint participation in the cell processes. SETX with DROSHA, XRN2, and RRP6/EXOSC10, can create a complex which causes premature transcription termination in lentiviral infection [68]; DROSHA—gene encodes ribonuclease (RNase) III (double-stranded RNA-specific ribonuclease); TAF4 (alias TATA-Box)—binding protein associated factor 4. TAFs may participate in basal transcription, serve as coactivators, function in promoter recognition, or modify general transcription factors (GTFs) to facilitate complex assembly and transcription initiation. TAF4, as a transcriptional cofactor of SETX, can reduce transcription activation by premature transcription termination. RRP6/EXOSC10—exosome component 10 has 3′-5′ exonuclease activity; HIV-1—human immunodeficiency virus 1; XRN2—gene encodes ribonuclease (RNase) III (double-stranded RNA-specific ribonuclease); SETX and XRN2, regulate RNA polymerase II pausing and premature termination and RRP6 generates a small RNA that represses transcription.

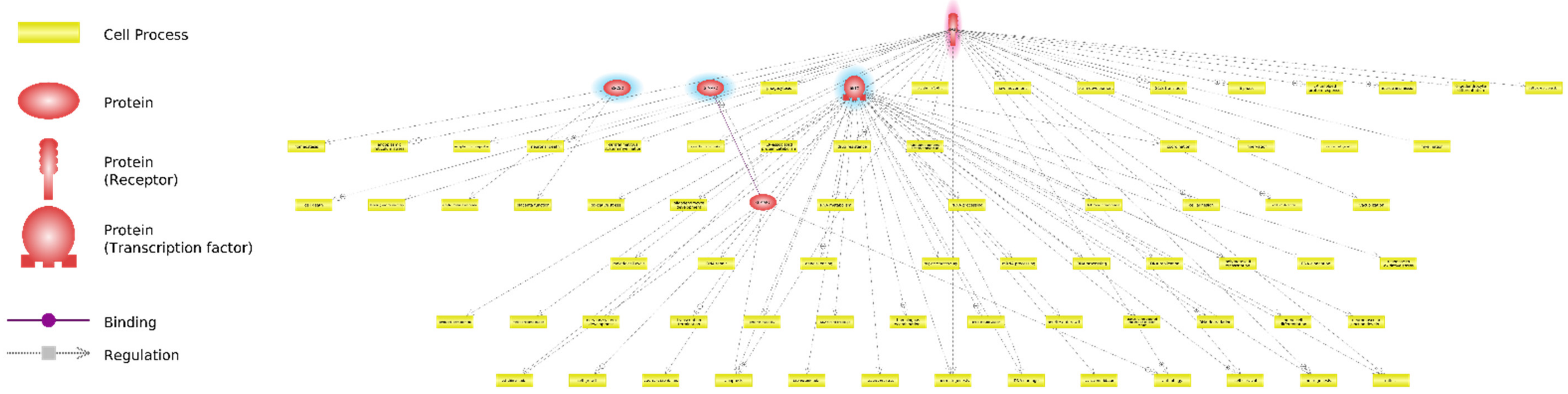

Figure 2. Hierarchical arrangement of the *SSC5D*, *GIMAP2–GIMAP7* dimer, *GPR37*, and *SETX* genes and their involvement in the regulation of cellular processes.
